# Presence of *Zea luxurians* (Durieu and Ascherson) Bird in Southern Brazil: Implications for the Conservation of Wild Relatives of Maize

**DOI:** 10.1371/journal.pone.0139034

**Published:** 2015-10-21

**Authors:** Natália Carolina de Almeida Silva, Rafael Vidal, Flaviane Malaquias Costa, Magdalena Vaio, Juliana Bernardi Ogliari

**Affiliations:** 1 Departamento de Fitotecnia, Centro de Ciências Agrárias (CCA), Universidade Federal de Santa Catarina (UFSC), Florianópolis, Santa Catarina, Brasil; 2 Departamento de Biología Vegetal, Facultad de Agronomía, Universidad de la República, Montevideo, Uruguay; Henan Agricultural Univerisity, CHINA

## Abstract

Records of the occurrence of wild relatives of maize in South American lowlands are unprecedented, especially in sympatric coexistence with landraces. This fact is relevant, because regions of occurrence of wild relatives of cultivated plants should be a priority for conservation, even if they do not correspond to the center of origin of the species. The aim of this study was to identify and characterize the wild relatives of maize in the Far West of Santa Catarina, southern Brazil. Therefore, phenotypic characterization was performed for five populations, based on 22 morphological traits deemed as fundamental for classifying the species of the genus *Zea*, and validated through the characterization of chromosomal knobs of two populations. The occurrence and distribution of teosinte populations were described through semi-structured interviews applied to a sample of 305 farmers. A total of 136 teosinte populations were identified; 75% of them occur spontaneously, 17% are cultivated populations, and 8% occur both ways, for the same farm. Populations that were characterized morphologically had trapezoidal fruits mostly, upright tassel branch (4–18), non-prominent main branch and glabrous glumes, with two protruding outer ribs and 8 inner ribs, on average. Cytogenetic analysis identified 10 pairs of homologous chromosomes (2n = 20) with 26 knobs, located in the terminal region of all chromosomes. The similarity of these results with the information reported in the literature indicates that the five populations of wild relatives of maize in this region of Santa Catarina belong to the botanical species *Zea luxurians*.

## Introduction

Wild relatives of cultivated plants include ancestral plant species, and other species that are related to a greater or lesser extent and share the same gene pool of the cultivated species [1–4]. They are important genetic resources because they comprise gene reservoirs for resistance to diseases, pests and extreme weather conditions such as drought, floods and cold. Moreover, they have important nutritional components [5,6,7].

For many farming communities, wild relatives contribute directly to food sovereignty and food security through the provision of fruits, leaves, fodder, tubers and seeds [8]. Although wild relatives are important, there are few efforts to preserve them because there is a limited number of protected areas, low representation in gene banks, and lack of information about their geographical distribution and genetic potential.

Wild relatives of maize comprise species of genera *Tripsacum* and *Zea*, the latter being commonly called teosintes. Teosintes are the closest wild relatives to maize. They are represented by diploid annual species, and perennial diploids and tetraploids [9–12].

There are two classifications for teosintes: the first is based on geographical distribution and ecological conditions [13,14,15]. Based on these aspects, five Mexican races of teosintes (Nobogame, Mesa Central, Chalco, Jalisco and Balsas) and two other Guatemalan races (Guatemala and Huehuetenango) have been described [9–15]. The second, more up-to-date classification, is based on taxonomic principles, which proposes the division of genus *Zea* into two sections: *Luxuriantes* and *Zea* [9,14].

The *Luxuriantes* section includes the species *Zea perennis* (Hitch) Reeves and Mangelsdorf, *Zea diploperennis* Iltis, Doebley and Guzman, and *Zea luxurians* (Dirieu and Ascherson) Bird; there was later inclusion of the species *Zea nicaraguensis* Iltis and Benz, in 2000. The *Zea* section includes *Zea mays* L., which is further divided into four subspecies: *Zea mays* subsp. *mexicana* (Schrader) Iltis, races Chalco, Mesa Central and Nobogame; *Zea mays* subsp. *parviglumis* Iltis and Doebley, races Balsas and Jalisco; *Zea may*s subsp. *huehuetenangensis* (Iltis and Doebley) Doebley, race Huehuetenango, and; *Zea mays* L. subsp. *mays*, which includes hundreds of cultivated maize races [9–15]. Molecular studies support the idea that the species *Zea mays* subsp. *parviglumis* is the progenitor of cultivated maize [16,17].

The geographical distribution of teosintes is restricted to tropical and subtropical areas of Mexico, Guatemala, Nicaragua and Honduras [12,13,18]. This distribution is not uniform and is closely related to climate, soil and specific crop-related aspects of each region [18]. However, most species are represented by only a few plant populations [14], or even by a few individuals. This shows that information on their geographical distribution is scarce [19], especially in regions outside the centers of origin.

In Brazil, the classification of maize races from South American lowlands by Goodman & Paterniani [20] is considered the main reference on maize germplasm in the country; however, it has no reports on the presence of teosintes in the Brazilian territory.

The earliest records of the presence of wild relatives of maize in Brazil were initially described by Pio Correa, in the 1930's work *Dicionário de Plantas Úteis do Brasil* ("Dictionary of Useful Plants in Brazil") [21]. In this publication, teosinte is commonly called *Guatemalan teosinte*, *Venezuelan grass* and *imperial grass* and classified as *Euchlaena mexicana*. Latest records date back to 1972 and describe the forage potential of teosintes, referred to as *milhina* and *dente de burro*, but they do not describe the origin of the species [22].

In Brazilian *ex situ* collections, the oldest accession of teosinte dates back to 1936 and is located in the germplasm bank of the *Instituto Agronômico de Campinas* (“Agronomic Institute of Campinas—IAC”), in the state of *São Paulo*. Passport data only contain the common name (teosinte), and the place of origin is described as *production fields in the state of São Paulo*. Overall, IAC has three teosinte accessions, one of 1937, called *florida teosinte*, a common name of the species *Zea luxurians* [23]; another is called *perennial teosinte*, also of 1937 and; *teosinte* of 1942. There is no information about the origin of the three accessions. In the germplasm bank of the *Empresa Brasileira de Pesquisa Agropecuária* (“Brazilian Agricultural Research Corporation—EMBRAPA”), eight teosinte accessions are preserved: six belong to the botanical species *Zea mays* subsp. *mexicana*, and one of them is called *dente de burro*; one belongs to the species *Zea diploperennis*, and one is *Zea perenni*s. All eight accessions were donated by the International Maize and Wheat Improvement Center (“CIMMYT”) in 1989 and 2011. There are no accessions collected in southern Brazil in either of the national institutions (IAC and EMBRAPA).

The first field observations about the presence of wild relatives of in the Far West of the state of *Santa Catarina* (SC), in southern Brazil, were performed in 2011, while a research study called *Census of Diversity* [non-published data], which was conducted by the *Núcleo de Estudos em Agrobiodiversidade* (“Center for Studies in Agricultural Biodiversity—NEABio”) at the *Universidade Federal de Santa Catarina* (“Federal University of *Santa Catarina*–UFSC”). In this study, 2,049 farmers from the municipalities of *Anchieta* and *Guaraciaba* were interviewed. A total of 1,513 maize landraces were identified and mapped; they were conserved *in situ/on farm*. The occurrence of wild relatives of maize was just observed and recorded and no other analyses were made, given the scope of the study on that occasion. Therefore, the aim of this study was to identify and characterize populations of wild relatives of maize in this region of the state of *Santa Catarina*.

## Material and Methods

### Local Knowledge on Wild Relatives of Maize

**Fig 1:**
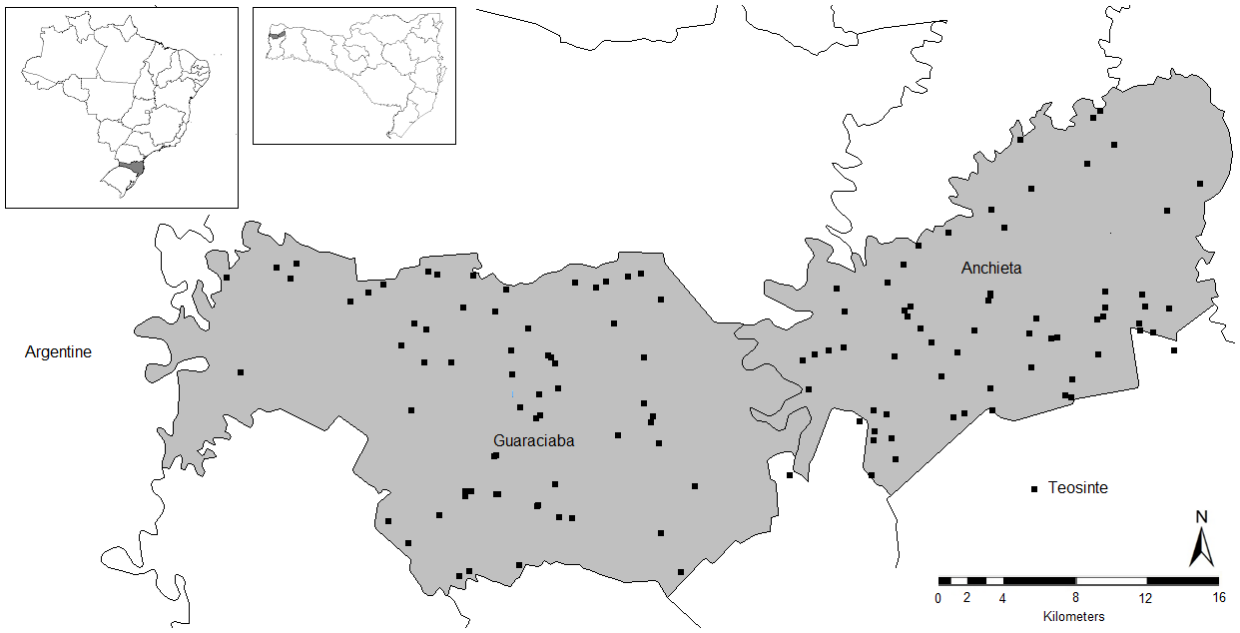
Location of the study area and geographic distribution of 136 teosinte populations. Map contour constructed from spatial data retrieved from http://www.diva-gis.org/gdata.

The characterization of the presence of wild relatives of maize was carried out through semi-structured interviews, guided by the form *Descriptors for farmers’ knowledge of plants* [24], with modifications to fit the context of the study area. The field research was carried out from January to July 2013, in the same municipalities covered by the *Census of Diversity* (Fig 1).

The database generated by the *Census of Diversity* was used to define the number of farmers to be interviewed. Only farmers who conserve local varieties of maize (total of 828) were selected. The sample universe was defined by the stratified sampling strategy proportional to the total number of farmers distributed by farm size (up to 5 ha, 5.01 to 10 ha, 10.01 to 15 ha, 15.01 to 20 ha, 20.01 to 30.0 h and greater than 30.01 ha). The sample consisted of 305 farmers, and it was based on the response variable; 50% proportion, 5% margin of error and 95% probability.

For each variable, exploratory analysis and inference of data were performed with descriptive statistics, using the number of observations whose data was complete. Mapping and geographical distribution (geographic and UTM coordinates) were performed with the geographic information system DIVA-GIS [25].

The field studies did not involve endangered or protected plant species; for this reason, no specific permissions were required for these locations and study field. They were carried out on private lands and the owners of the lands gave permission to conduct the study on these sites. Written consent was given by local organizations that represent farmers (Small-Scale Farmers’ Trade Union—SINTRAF, Association of Local Maize Variety Farmers and Processors—ASSO, and Porerekan Institute) and also individually by 305 interviewed farmers. In order to characterize local knowledge on teosintes, semi-structured interviews were applied; the data was analyzed anonymously.

### Plant Material and Morphological Descriptors

The genetic material included in this study consisted of five teosinte populations collected in farms in the municipalities of *Anchieta* and *Guaraciaba* (Table 1) in the Far West of the state of *Santa Catarina*, depending on availability and accessibility of seeds at the time of the survey; written consent also was given for the characterization of these populations. Data was collected in July 2013, and the germination test was applied according to the international protocol [26].

**Table 1:** Location of collection areas of five teosinte populations.

Population	Municipality	Collection area	Altitude (meters)	Latitude	Longitude
T2484	Anchieta	Grassland	501	-26.6237	-53.4949
T2335	Anchieta	Grassland	619	-26.4860	-53.2530
T824	Guaraciaba	Maize crop	594	-26.5444	-53.6103
T51	Guaraciaba	Maize crop	570	-26.5972	-53.3976
T2021	Anchieta	Grassland	696	-26.5731	-53.3047

Morphological characterization was performed at *Centro de Ciências Agrárias* (“Center for Agricultural Science—CCA”) at UFSC, located in *Florianópolis* (altitude 2.0 meters), from September 2013 to May 2014. Each population was assessed in a row of 4.0 meters in length, spaced one meter between rows. Data was collected from nine or ten plants of each population, with subsequent herborization of the material for botanical identification of the species.

A total of 18 quantitative and four qualitative morphological descriptors were assessed. Quantitative descriptors were male flowering (days), female flowering (days), mean number of leaves per plant, mean length and width of flag leaf, mean number of tillers per plant, mean number of side branches, mean plant height (cm), mean height of lower ear (cm) and mean height of upper ear (cm), mean number of total tassel branches, tassel length (cm), length of main tassel branch (cm), mean length of outer glume (mm), mean width of outer glume (mm), mean number of ribs of outer glume, mean length of upper ear (cm) and hundred-seed weight (gr). The evaluated qualitative descriptors were shape of tassel branch (lax or upright), type of main tassel branch (prominent or non-prominent), presence of the abscission layer of tassel branches, shape of outer glume, curvature of outer or inner glume, presence of ribs on the sides of glumes, shape of grain capsule. Glume traits were evaluated with a magnifying glass and a digital camera. The evaluated descriptors are considered key to the botanical identification of species of the genus *Zea* [9,10]; most of them were also studied by several authors [18,27].

Mean values of each population were estimated for each variable. The total values of the population were collected for the variables male flowering, female flowering and hundred-seed weight. Principal component analysis (PCA) was performed through the R package mixOmics [28]. The variables male flowering, female flowering and hundred-seed weight were not included in PCA.

### Cytogenetic Characterization for Validation of Species

Cytogenetic characterization was performed to validate the results found by morphological characterization and botanical identification. The analyses were performed at the *Departamento de Biología Vegetal* (“Department of Plant Biology”), *Facultad de Agronomía* (“Agronomy School”) of *Universidad de la República* (“University of the Republic”) in Uruguay.

Mitotic analysis was performed with root tip from seeds germinated in a Petri dish or from plants maintained in pots at the greenhouse. Roots were pretreated with 0.002 M 8-hydroxiquinoline at 10°C for 20–24 h, fixed in Carnoy 3:1 (ethanol/glacial acetic acid) for 2 to 24 h at room temperature and then stored at -20°C. For cytological preparations, roots were digested with a mixture of 2% cellulase and pectinase 20% (w/v) for three hours at 37°C, then treated with 60% acetic acid for at least 30 min, and then the meristem was dissected and squashed in 45% acetic acid. After removal of the coverslip with liquid nitrogen, slides were air dried and aged for three days at room temperature.

Double staining with fluorochromes chromomycin A3 (CMA) and 4,6-diamidino-2-phenylindole (DAPI) was performed according to the protocol described in Cabral et al. [29]. Aged cytological preparations were stained with 0.1 mg/ml CMA for an hour and 1 μg/ml DAPI for half an hour. Finally, they were mounted in 1: 1 (v/v) McIlvaine's buffer solution—pH 7/glycerol.

The best images of cells were collected using a Leica DMLB microscope fitted with a Cohu CCD camera and Leica QFISH software. The images were processed with Adobe Photoshop CS3 software only for brightness and contrast. Karyograms and measurements were conducted in the same software. Software Corel Draw X6 was used to design the ideogram.

## Results

### Spatial Distribution and Farmers' Perception of Teosinte Populations

A total of 136 teosinte populations were identified and mapped (Fig 1). In both municipalities, most farmers (96%) refer to teosinte as *dente de burro*, a term used locally to identify teosintes. Spatial distribution was not restricted to the study area only. Farmers reported seeing *dente de burro* in at least 12 other municipalities in the state of *Santa Catarina* and also in the southern states of *Paraná* and *Rio Grande do Sul*.

According to farmers' accounts, teosinte populations have been present in the region at least since 1949. This is the earliest date mentioned in the interviews. This information coincides with the origin of the 136 populations identified in this survey: 31% were bought locally, with seeds being informally produced by farmers themselves and sold to agricultural supply stores; 26% of them were cited as pre-existing on the farms; 21% were introduced when the family migrated from *Rio Grande do Sul* to *Santa Catarina*; 12% were bought from neighboring farmers; 6% of the farmers did not remember the origin and; 4% had other sources, such as dispersal by animals and agricultural machinery.

Teosinte plants were described by farmers as being annual and having distichous ears; mean plant height and number of tillers per plant were estimated at 2 meters and 10 tillers, respectively. The character that showed the greatest variation was grain color, with at least seven cited shades: gray (32%), multicolored (26%), white (18%), brown (17%), black (4%), yellow (2%) and purple (1%).

The presence and distribution of the species in the region are related to its use for grazing, especially by dairy cattle. Forage potential was mentioned by 43% of farmers and other 10 characteristics were mentioned, whether or not associated with its main use. There was a total of 388 mentions of pest resistance (35%), disease resistance (27%), drought tolerance (16%), milk yield (9%), grassland yield (8%), grassland softness (4%), tolerance to high temperatures (3%), tolerance to herbicides (2%), and other mentions (1%), such as tradition and craftsmanship and tolerance to low temperatures (0.7%).

The farmers’ management practices resulted in the two categories of teosinte populations: *spontaneous* (75% of the population observed), *cultivated* (17%) and, to a lesser extent, both types (8%). *Cultured* populations are those in which farmers save seeds in every generation to the next harvest, which occurs between the months of August and November, the same time of maize planting in the region. A fraction of the grassland area is intended for producing seeds whose harvest is performed before the seed dispersion process. Farmers consider *spontaneous* populations as those that grow undesirably in maize, sugar cane and cassava crops, in other grassland areas and on riverbanks. The frequency of these populations was considered to be high, as they occur every year during the hot and rainy season in the region (between August and December).

The presence of *spontaneous* populations is due to the fact that many farmers have abandoned the cultivation of teosinte in order to introduce summer oats (*Sorghum sudanense* L.) for grazing purposes. In addition, the seeds are dispersed by birds, farm machinery and cattle, which graze on areas contaminated or cultivated with teosintes and defecate in other areas without previous occurrence of this species. Seed dispersal interfere in farmers' management; 54% of them said they establish some kind of control to prevent spread to other areas of the farm.

Evidence of gene flow with formation of natural hybrids between maize and teosinte, in both directions, was mentioned by 49 farmers (S1–S2 Figs). These accounts are based on field observations that show changes of morphological characteristics such as: (i) forming of ears with four-grain rows, larger yellow grains and grains whose endosperm is similar to that of popcorn, in teosintes, and; (ii) narrowing of leaves, tillering of cobs, formation of grainless ears, and grain expansion capacity in maize, whose endosperm is not similar to that of popcorn.

Gene flow is favored by the occurrence of *spontaneous* populations in areas cultivated with maize, by the proximity of grazing land to maize crops and coincidence in the planting season.

### Morphological Characteristics

Table 2 shows the results of morphological descriptors used to characterize the five teosinte populations. All populations predominantly had upright tassel branch, non-prominent main branch and glabrous glumes. The male glumes had prominent (outer) side ribs and 6–18 inner ribs, with an average of 8 ribs. The number of tassel branches ranged from 4 to 18. The populations T2021, T2335 and T2484 showed predominantly trapezoidal fruits, while T51 and T824 populations showed predominantly triangular fruits (S3–S7 Figs).

**Table 2:** Mean values of morphological characteristics of five teosinte populations.

	Populations					
	51	824	2021	2335	2484	Standard Deviation
	Number of evaluated individuals per population					σ
**Morphological characteristics**	10	9	10	10	9	
Male flowering (days)	149	129	171	158	133	**15.6**
Female flowering (days)	173	161	181	175	157	**9.0**
Total number of leaves	16.3	18.2	15.2	14.0	14.1	**1.6**
Flag leaf length (cm)	27.0	54.0	37.2	29.2	38.7	**9.5**
Flag leaf width (cm)	3.1	6.0	5.3	3.9	5.3	**1.1**
Number of tillers	4.8	2.4	4.9	5.6	8.5	**2.0**
Number of side branches	3.6	4.2	4.7	3.2	4.5	**0.6**
Plant height (m)	2.7	2.7	2.9	2.5	2.6	**0.1**
Lower ear height (m)	1.6	1.5	1.8	1.4	1.6	**0.1**
Upper ear height (m)	2.0	1.8	2.3	1.9	2.0	**0.2**
Upper ear length (cm)	14.3	9.2	12.2	13.4	12.7	**1.7**
Number of tassel branches	14.0	10.3	10.7	11.0	12.4	**1.4**
Tassel length (cm)	20.0	28.9	21.7	20.8	23.1	**3.2**
Length of main tassel branch (cm)	11.9	19.6	13.7	15.2	14.1	**2.6**
Glume length (mm)	9.6	11.4	9.8	9.2	9.3	**0.8**
Glume width (mm)	2.7	2.9	2.8	2.7	2.6	**0.1**
Number of glume ribs	8.7	10.9	9.6	9.7	11.3	**0.9**
Thousand-seed weight (g)	77.1	88.8	81.2	68.2	86.4	**7.3**

The characteristics that showed the least variation between plants within populations were number of side branches (4.0); plant height (2.70 m); lower ear height (1.6 m); upper ear height (2.0 m); glume length (9.9 mm); glume width (2.7 mm) and number of glume ribs (10.0). The characters that showed the greatest variation between plants within populations were flag leaf length (37.2 cm); tassel length (22.9 cm) and length of main tassel branch (14.9 cm).

**Fig 2:**
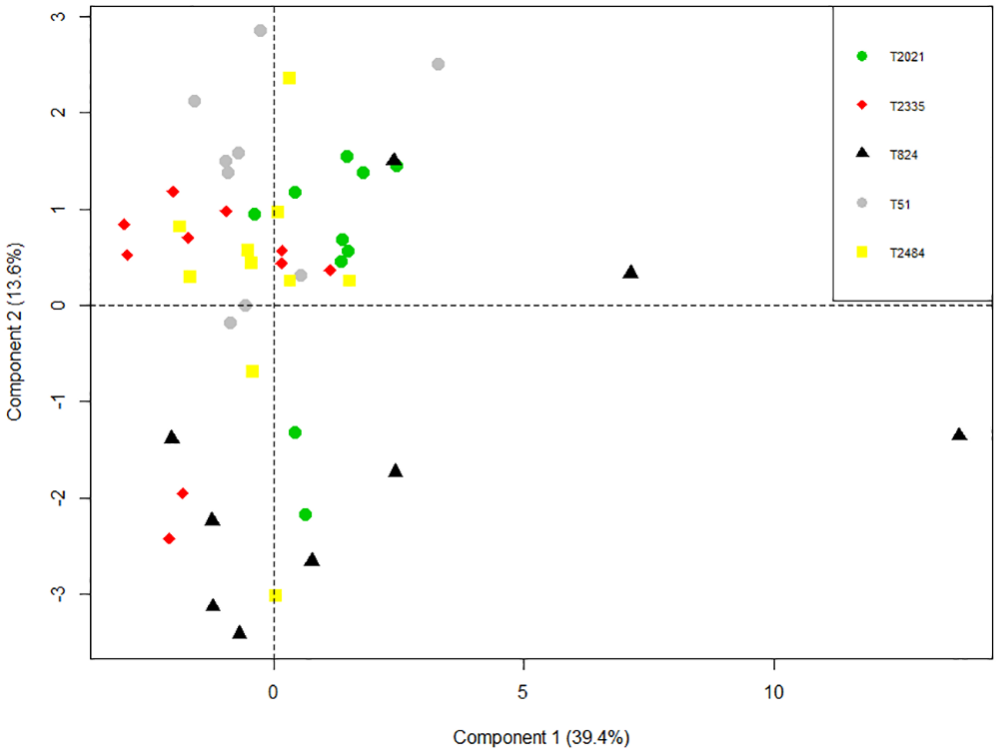
Principal components of 15 quantitative morphological variables for five teosinte populations.

The exploratory analysis of principal components (Fig 2) was performed in order to identify divergence between populations. The first axis explained 39.4% of the variation and the second axis, 13.6%. The analysis suggests that all populations have individuals distributed in the first quadrant, and most individuals are distributed in the first and second quadrants.

The T824 population showed greater dispersion on axis one, and individuals are predominantly distributed in the third and fourth quadrants. This result is explained by the fact that two individuals have the highest values for leaf length and width. These characteristics were highly variable and highly correlated (0.88 and 0.72, respectively) with axis one.

### Chromosome Number and Heterochromatic Blocks

**Fig 3:**
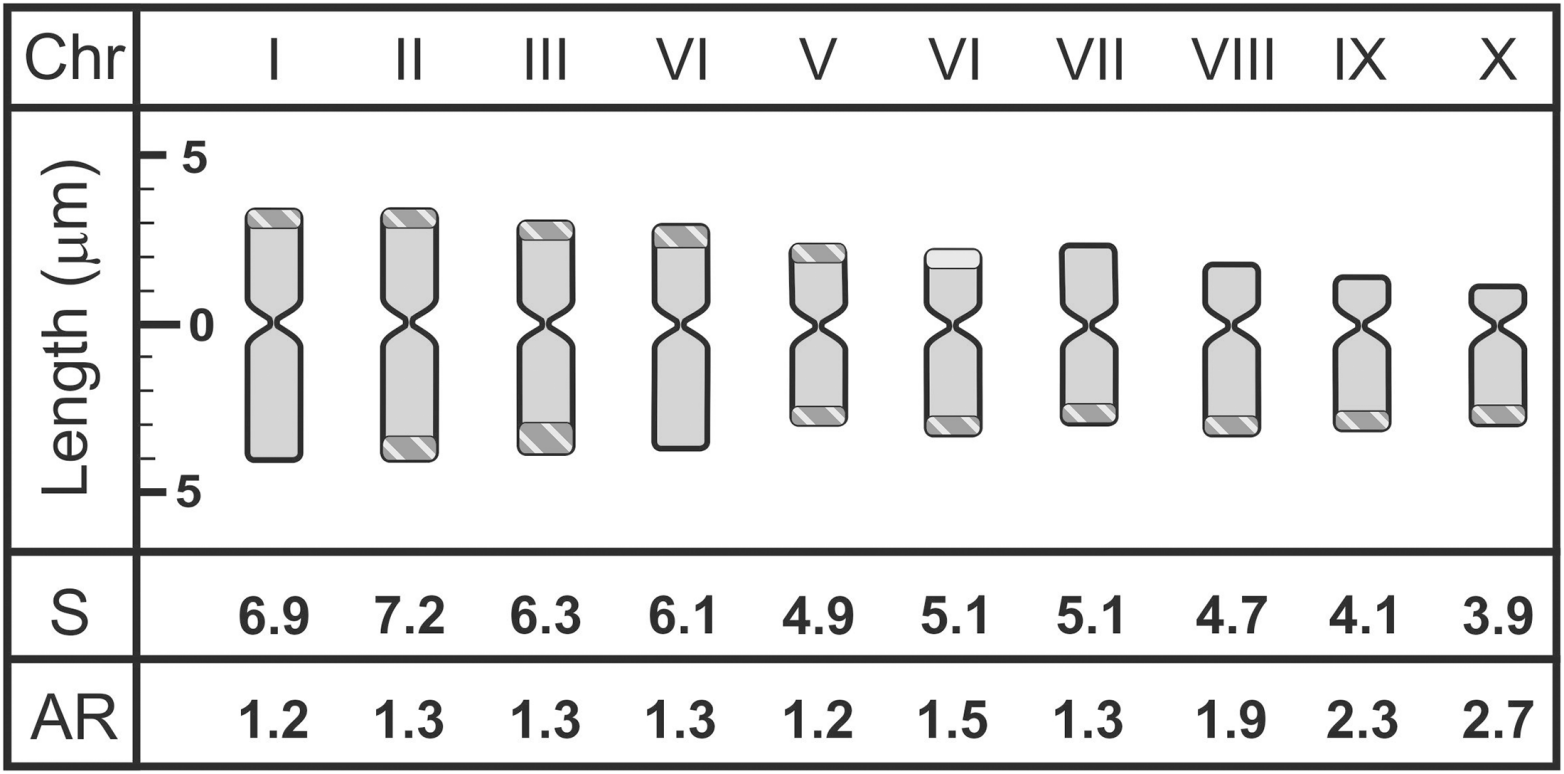
Idiogram representation based on chromosome size, ratio between chromosome arms and patterns of heterochromatic bands. Chr: identification of 10 chromosomes; S; chromosome size; AR: ratio between chromosome arms.

The two populations showed a chromosome number of 2n = 20. Chromosome size ranged from 3.9 to 7.2 μm (Fig 3) and total size of the chromosomal complement was approximately 110 μm. The karyotype was symmetrical and consists of four pairs of metacentric chromosomes (Pair 1–4), five submetacentric (Pair 5–9) and an almost acrocentric pair (Pair 10; ratio between arms = 2.7). Fig 3 shows the idiogram, chromosome sizes and ratios between arms. The chromosomes were arranged according to the karyogram proposed by González et al. [30] on the basis of DAPI^+^ bands.

**Fig 4:**
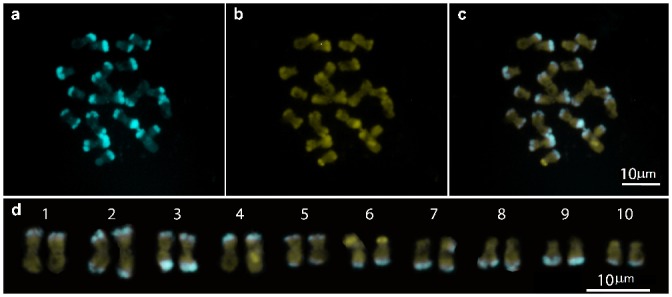
Location of chromosomal knobs. Heterochromatic DAPI+ bands located in the terminal regions of the chromosomes.

All chromosomes showed DAPI- bands, totaling 13 pairs of DAPI^+^ bands and located in the terminal regions of chromosomes (Fig 4). The chromosome pairs 1 and 4 showed a terminal DAPI^+^ band in the short arm, while the pairs 2, 3 and 5 showed DAPI^+^ bands at telomeric regions on both chromosome arms. In the other chromosomes, the DAPI- band was observed in the telomeric region of the long arm. Different intensities were observed between bands; those located on the long arm of chromosome pairs 3 and 9 were the most intense and probably had more copies of the repeat sequences that conform them.

All DAPI ^+^ bands were also stained with CMA (CMA^+^ / DAPI^+^ bands), although with less intensity. The only bands that were CMA^+^ but not stained with DAPI (CMA^+^ / DAPI°) are located in the terminal region of the short arm of chromosome 6, probably coinciding with the NOR region.

## Discussion

### New Populations of *Zea luxurians* in Southern Brazil

Teosinte populations in Far West of *Santa Catarina* are characterized by having male glumes with two prominent (side) outer ribs, abundant internal ribs, scarcely branched tassel and predominantly trapezoidal fruits, with the exception of T51 and T824 populations, whose fruits are predominantly triangular. These results coincide with the characterization performed by Doebley and Iltis [10] for the species *Zea luxurians*. This species is characterized by having male glumes with two prominent (side) outer ribs, with 9 to 24 internal ribs, tassel with 9 to 24 branches (10 on average) and trapezoidal fruits [10].

The features number of leaves and 100-seed weight coincide with those found by Sánchez et al. [18] for *Zea luxurians* populations of Guatemala, with 15 and 85 g, respectively. The characters female flowering (166 days), male flowering (146 days), tassel branch number (16) and number of branches of main stem (average 3.7) were coincident with the characteristics described by Sánchez et al. [18] for *Zea nicaraguensis*. Tassel branch number was also matched to *Zea luxurians* populations of Guatemala when compared with studies by Loáisiga et al. [31]. Data from molecular markers show that the species *Zea nicaraguensis* and *Zea luxurians* are closely associated [32].

The T824 and T51 populations are the only *spontaneous* ones. They were collected in maize fields and had larger and yellow grains. Occasional crosses with maize could explain the predominance of triangular grains.

Sánchez et al. [18] also identified morphological differences between populations of the same species of the section *Luxuriantes* from different regions. However, principal component analysis (Fig 2) shows that, despite their differences, these populations belong to the same group and have some disparate individuals.

Chromosome heterochromatin blocks or knobs have been widely studied and characterized in the different taxa of the genus *Zea* and occur in all species with 2n = 20. The position and number of knobs vary between different strains, races and species of the genus and the observed patterns have been used by several authors to characterize, identify, classify and even suggest evolutionary relationships [33–37].

In maize, there are variations between populations and plants from the same population. Despite all variations, knobs can be used as taxonomic markers because they have a relatively fixed number and location within specific taxa, and they vary between different taxa [38].

All subspecies of *Zea mays* L. ssp. have interstitial knobs and few or no terminal knobs; however, in the species of the section *Luxuriantes*, all knobs are terminal. The largest number of heterochromatic bands is present in *Zea luxurians* and *Zea nicaraguensis*; the difference is that in the latter, a pair of chromosomes has no knobs [36]. *Zea luxurians* is probably the species within the genus with the most distinctive and conserved pattern of heterochromatic knobs. The two populations analyzed in this study had the same number and location of heterochromatic knobs and all chromosomes had at least one knob in terminal location.

These characteristics, as well as the high number of observed knobs (26 sites), coincide precisely with the heterochromatic patterns described for *Zea luxurians* by González & Poggio [30] González et al. [37] and Ellneskog-Staam et al. [36]. However, there are differences in the chromosome arms that carry the knob, as far as the latter authors are concerned, certainly, due to the fact that they conducted their studies with prometaphase chromosomes, with a different degree of condensation than the one in this study.

The observations made for the populations of the Far West of *Santa Catarina* and their exact resemblance to data previously described for the species leave no doubt that the populations correspond to the species *Zea luxurians*.

### The Introduction of Teosintes in Southern Brazil

Unlike maize, information about the migration and dispersion of teosintes is scarce. Generally, it is argued that official introductions into other countries have been carried out according to their forage potential. In fact, in the twentieth century, there have been many introductions into Southern United States, the Caribbean Islands, South America, India and Pakistan [39].

The species *Zea luxurians* is endemic to South Guatemala [12, 18, 23, 40], although it has also been reported in Oaxaca, Mexico [18]. In 1921, Collins had already reported as uncertain the origin of *florida* teosinte, which corresponds to the species *Zea luxurians*. According to this author, *florida* teosinte appears to be the same as the one distributed from France to many tropical countries, and the early records indicate that teosinte was introduced into France from Guatemala [41].

Given the above, the introduction of teosinte in southern Brazil is related to official introductions made by public research institutions, probably coincident with the beginning of the characterization of maize germplasm in Brazil by Brieger et al. [42] and later, more intensively, by Paterniani and Goodman [20]. This hypothesis can be supported by the passport data of accessions kept in national germplasm banks, which indicate the existence of several official introductions, some prior to 1930, which enabled the records of Pio Correa.

In southern Brazil, farmers in the Far West of *Santa Catarina* have been using teosinte as forage for at least 65 years. This period coincides with the colonization of the region that was intensified in the 1940s [43], with the migration of European descendants coming from the state of *Rio Grande do Sul* [44].

Teosinte has been probably introduced in southern Brazil precisely because of its potential for forage production, i.e., public research institutions were already aware of its possibilities of use and helped promote it for this purpose. Generally, official introductions of a particular germplasm with a specific use, allow its rapid spread to various regions, maintaining the same original name. This claim is convergent with the fact that 90% of farmers refer to teosinte as *dente de burro*.

### Gene Flow Evidence between Maize and Teosinte

Gene flow is one of the mechanisms of diversity and evolution of species. In maize and teosinte, pollen is responsible for the flow of genetic information across populations that coincide or overlap in time and space [45]. This is an ongoing process, in which the wide distribution of maize crops may help connect different populations, including allopatric ones [46]. Gene flow in the genus *Zea* has been well documented not only by the presence of hybrids in F1 populations [11, 47] but also by molecular studies [14, 48]. Evidence of genetic contribution of teosintes into the gene pool of 75 corn landraces was observed in studies performed by Warburton et al. [49]. On artificial crosses between maize and *Zea luxurians*, Molina et al. [50] found that 89% of the seeds were fertile. The characteristics of hybrid individuals were height between 2 to 3 meters, over ten tillers per plant, ears with dehiscent grains and photoperiodic control of flowering.

In the Far West of *Santa Catarina*, F_1_ hybrid plants were identified between teosinte and maize, both by reports of farmers and seed collection (S1–S2 Figs). The perception of farmers concerning the gene flow is supported by the fact that populations occur mainly in maize fields, and also because the planting season of cultivated populations coincides with the time of maize planting in the region.

Within the T824 population, whose seed collection was carried out in a maize field, hybrid individuals were observed to have yellow grains. The germination percentage of this population was 87%, and this result coincides with information reported in the literature [50], which shows the gene flow between maize and *Zea luxurians*.

The presence of teosinte populations in sympatric coexistence with maize landraces and the evidence of gene flow between these species establish a new scenario for the Far West of *Santa Catarina*. In this survey, we found additional evidence of the presence of maize alleles into teosinte populations. Our discovery supports the argument that the gene flow between the two species, which belong the same gene pool, may be contributing to the evolution of both the populations in this region, although we did not have asked directly to the farmers if they used to select hybrid plants.

### Why Should Wild Relatives of Maize Be Conserved In Situ in Southern Brazil?

Brazil is not the “center of origin” of the species *Zea luxurians*, but it can be considered as a “country of origin”. This means that the country has this genetic resource *in situ* [51]. Both the Convention on Biological Diversity and the International Treaty on Plant Genetic Resources for Food and Agriculture, to which Brazil is a signatory, require that countries should promote *in situ* conservation of wild relatives of cultivated plants and wild plants, also in protected areas, by supporting the efforts of indigenous and local communities [51,52].

Together with international agreements, some other reasons justify the development of conservation strategies for wild relatives in this small geographical area of the Far West of *Santa Catarina*; together, the two municipalities (*Anchieta* and *Guaraciaba*) cover an area of 558.7 km^2^.

The first reason is the importance of this genetic resource for farmers. There were 388 indications of use for agronomic and adaptive values, as perceived by farmers. This demonstrates that not only one, but also a set of characteristics makes the species *Zea luxurians* interesting for the region.

The occurrence and conservation of *Zea luxurians* populations are important for the production of milk, which is the main economic activity in the region. The species persists because it is used and, at some point in history, it was introduced in this region, hence it became useful for the production strategies of farmers.

This was also described by other authors for different species, especially in the studies performed by Benz et al. [53] for *Zea diploperennis*, in the context of the Manantlán Reserve in Jalisco, Mexico, by Miranda for *Zea mays* subsp. *parviglumis* [54], and by Vibrans and Estrada for *Zea mays* subsp. *mexicana* [55].

Each species or variety has a particular significance for farmers, and use values motivate farmers to keep some populations in preferred locations, i.e., on their farms [56]. Thus, local crops tend to disappear if farmers do not grow them for some reason or do not have some sort of value [56, 57].

These are precisely the use values identified for populations of *Zea luxurians*, the mechanism which depicts the conservation strategy of using the diversity in the region, which contributes to the local economy and at the same time, enables the *in situ*/*on farm* conservation of the species. Undoubtedly, describing the uses, the local nomenclature and the forage potential of teosinte, as well as its morphological characteristics and its distribution, is extremely important to provide conservation.

The second reason is related to the occurrence of new teosinte populations in geographical regions with climate and soil characteristics, topography and altitude that differ from those of their centers of origin. This factor can add further knowledge about the gene pool of the genus, which is considered the ancestor of one of the most important cereals in the world.

The populations of *Zea luxurians* in the Far West of *Santa Catarina*, whose presence in the region dates back at least 65 years, occur in isolation of the native populations of Central American and Mexico, whose climate and soil conditions and altitude are very different from those found in this region of Brazil. A differentiation is also expected between *Zea luxurians* populations in Central American and Mexico, which could even lead to the emergence of new races. In addition, gene flow between maize landraces and teosintes in southern Brazil may also contribute to generate unique diversity. However, further research should be conducted to compare morphological and genetic characteristics of *Zea luxurians* populations of Guatemala and Mexico with populations of *Santa Catarina* in order to confirm this hypothesis. With that established, *Zea luxurians* populations of this region of Brazil could be of interest to the *ex situ* conservation.

The third and final reason, and perhaps the most complex one, is the fact that regions spanning wild relatives and an important diversity of landraces should be determined as priority zones for agrobiodiversity conservation and, thus, genetically modified (GM) free zones. In this way, there are zones free of GM cotton plants in Brazil for the preservation of native or naturalized species of *Gossypium* [58]. The occurrence of *Zea luxurians* in the Far West of *Santa Catarina* is much older than the date of approval of the first GM maize event in Brazil, in 2007. However, this fact was not taken into consideration by national biosecurity standards.

Based on these facts, gene flow between maize and teosinte in this part of Brazil should be analyzed from the point of view of biosecurity. The expansion of cultivated areas with GM maize in this region, mainly of herbicide-resistant maize, may be a threat for the local agricultural ecosystems, considering the risks of gene flow to *spontaneous* teosintes that grow in GM maize fields. In fact, there were mentions of herbicide-tolerant teosinte (2%) by interviewed farmers as well as the occurrence of gene flow between maize and teosinte in this region. This scenario shows the fragility of the biosecurity standards that are based on establishing minimal distances between GM and non-GM maize fields [59] in order to guarantee the coexistence of both of them without contamination, since the teosinte may be serving as species-bridge for the undesirably occurrence of gene flow. In this sense, this study warns about the importance of considering the conditions of regional agroecosystems in order to elaborate safe agricultural policies for the agrobiodiversity conservation.

Future studies are still required to further investigate the phylogenetic relationships among teosinte populations in Brazil and in other countries, in order to check the level of diversity of this region, to identify whether there are erosion or evolution processes (or both), and to observe the frequency of gene flow between populations of *Zea luxurians* and maize. Further studies should be also performed to verify whether there are other species of teosintes in the target region.

## Conclusions

The populations of the Far West of *Santa Catarina* evaluated in this study belong to the botanical species *Zea luxurians*. Teosinte populations have been present in the region at least since 1949. They were introduced during the process of migration of European descendants, mainly from *Rio Grande do Sul* to *Santa Catarina*. They remained present in the region because they are used for forage purposes.

The presence of teosinte populations in sympatric coexistence, with maize landraces and the evidence of gene flow, establish a new scenario for the Far West of *Santa Catarina*, mainly as regards national policies of *in situ*/*on farm* conservation.

The discovery of new teosinte populations in geographical regions with climate and soil characteristics, topography and altitude different from those of their centers of origin, associated with the management carried out by farmers in this region, opens up new possibilities for discussion on the existence of a new race of *Zea luxurians* in Brazil.

## Supporting Information

S1 Fig Teosinte x maize hybrid. Ear characteristics: natural crossing between teosinte and landrace of maize named *Palha Roxa*. (TIF)

S2 Fig Teosinte x maize hybrid. Whole plant characteristics. (TIF)

S3 Fig Morphological characteristics of fruits of the T2021 population. (TIFF)

S4 Fig Morphological characteristics of fruits of the T2335 population. (TIFF)

S5 Fig Morphological characteristics of fruits of the T2484 population. (TIFF)

S6 Fig Morphological characteristics of fruits of the T51 population. (TIFF)

S7 Fig Morphological characteristics of fruits of the T824 population. (TIFF)
