# Supplementary figures and images for: Presence of Zea luxurians (Durieu and Ascherson) Bird in Southern Brazil: Implications for the Conservation of Wild Relatives of Maize
Source: PLoS One. 2015 Oct 21;10(10):e0139034. doi: 10.1371/journal.pone.0139034 (PMC4619479; doi:10.1371/journal.pone.0139034)

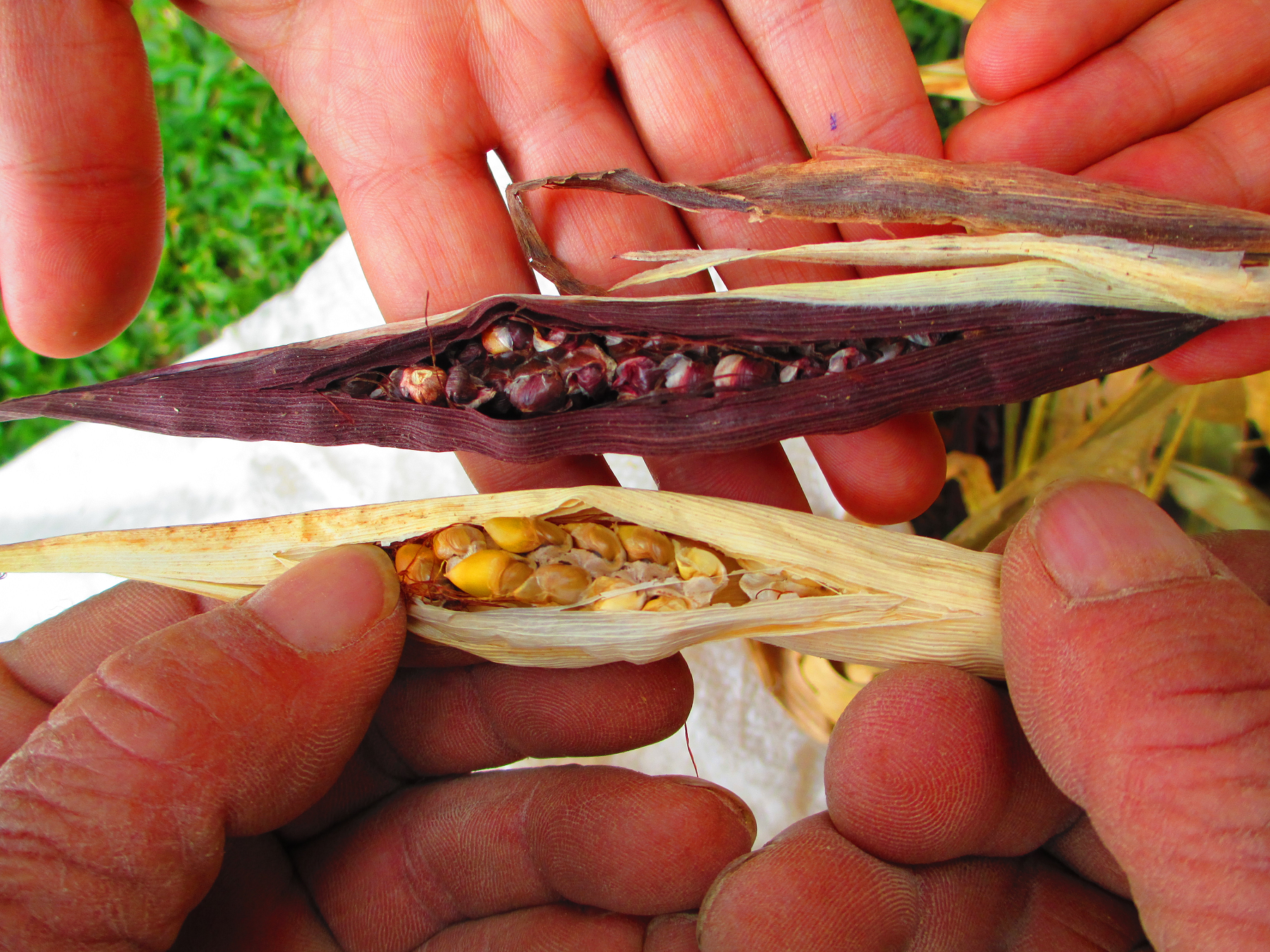

Supplement: S1 Fig — (TIF) [file pone.0139034.s001.tif]

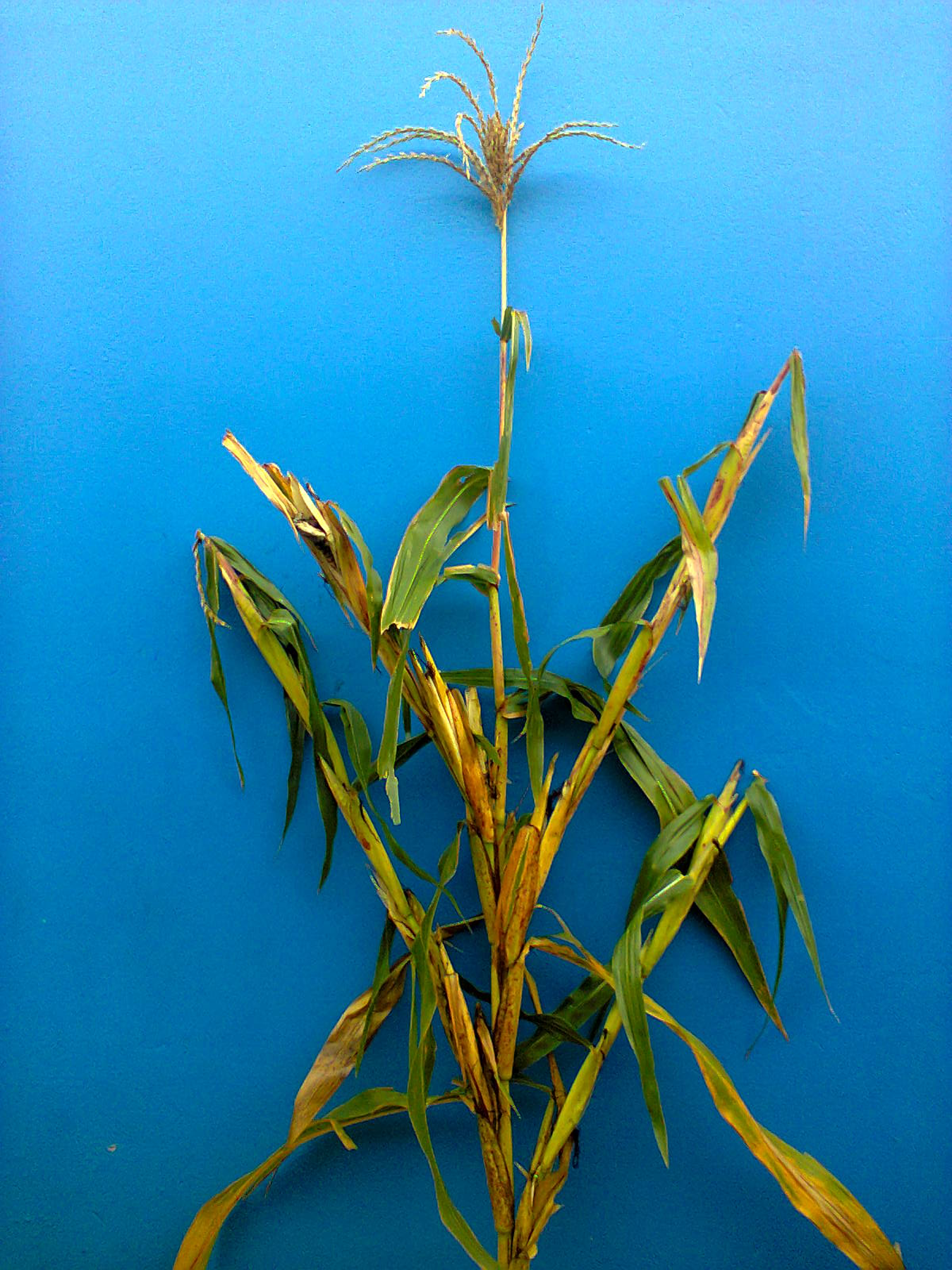

Supplement: S2 Fig — (TIF) [file pone.0139034.s002.tif]

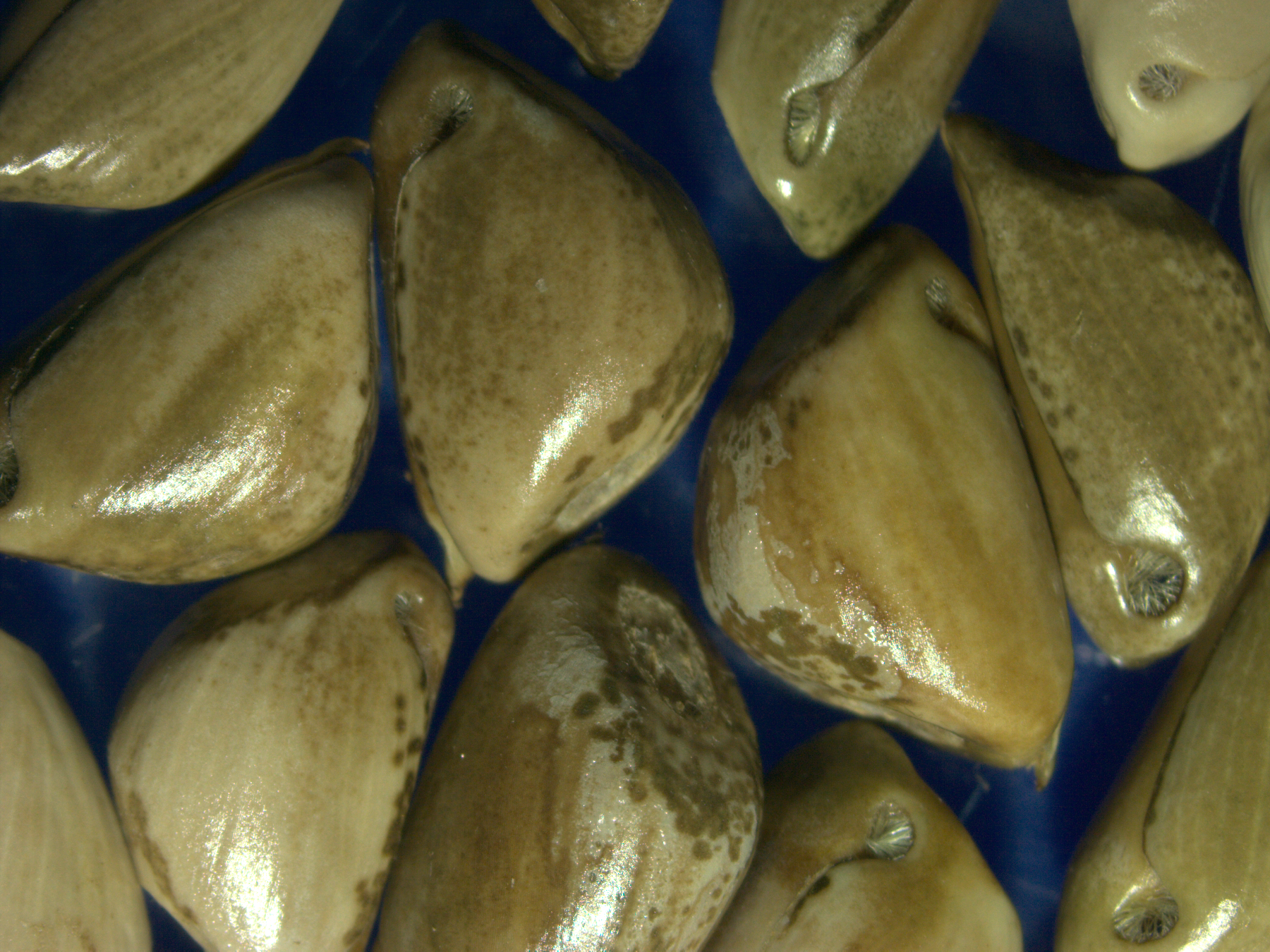

Supplement: S3 Fig — (TIFF) [file pone.0139034.s003.tiff]

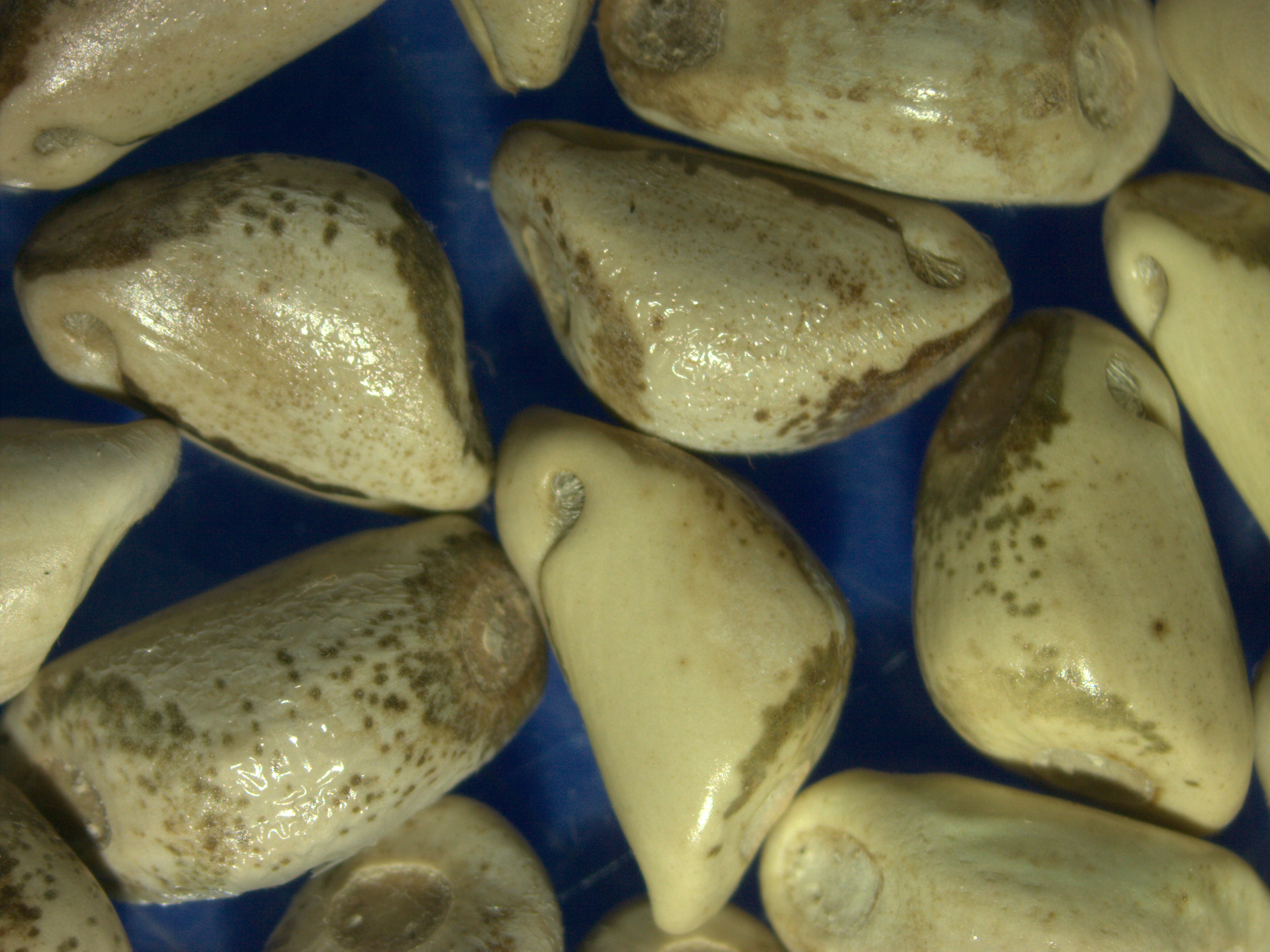

Supplement: S4 Fig — (TIFF) [file pone.0139034.s004.tiff]

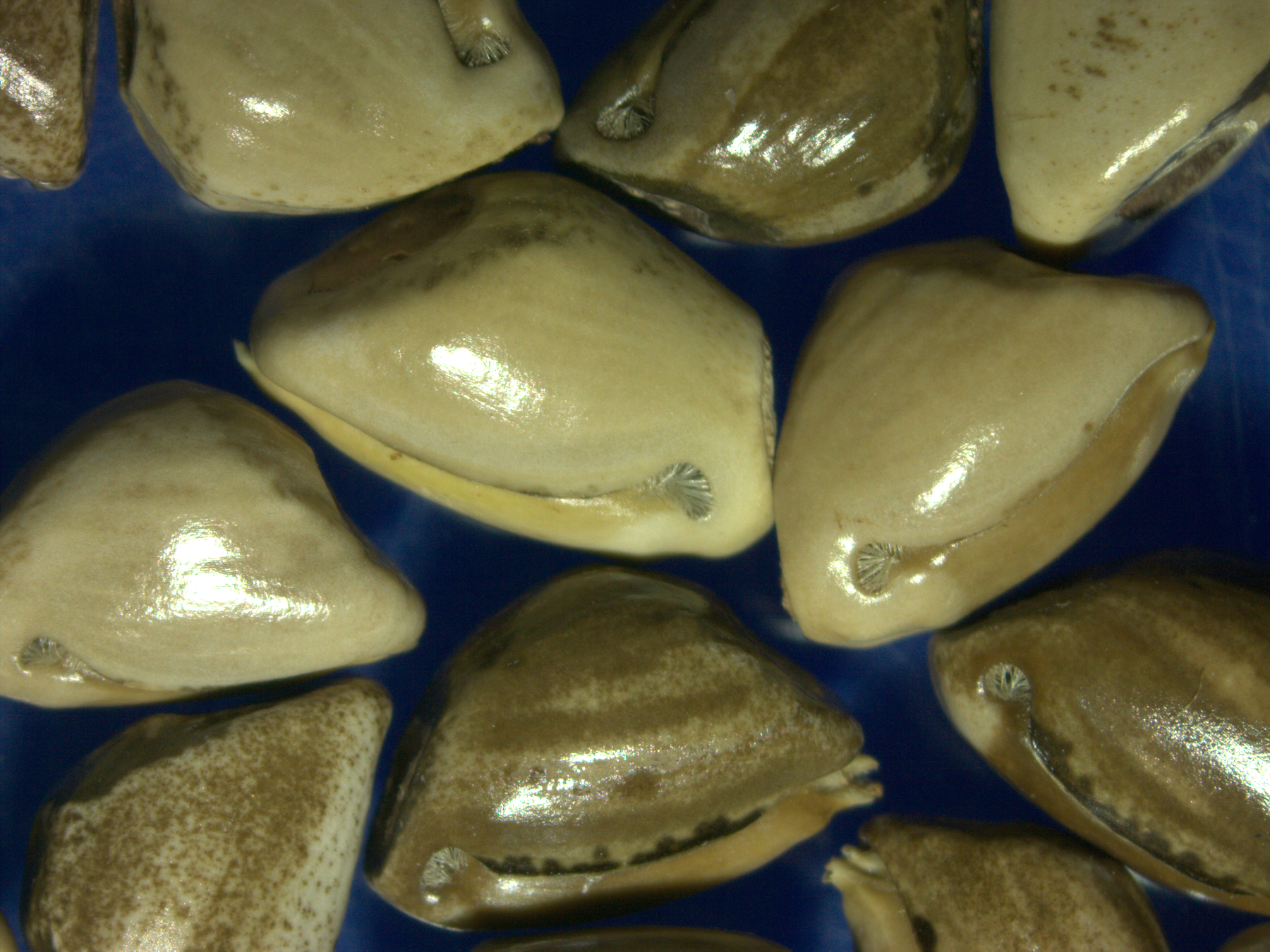

Supplement: S5 Fig — (TIFF) [file pone.0139034.s005.tiff]

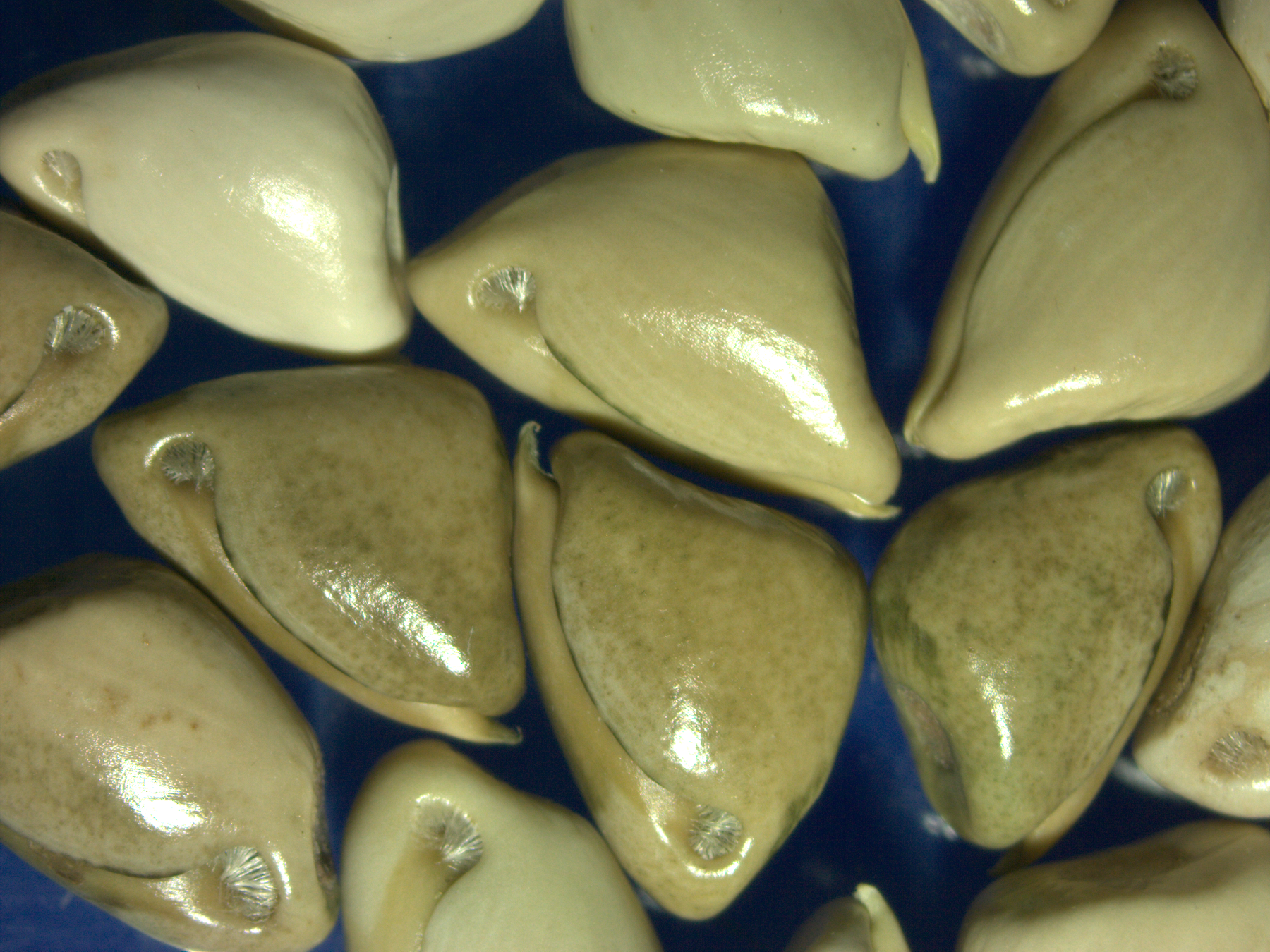

Supplement: S6 Fig — (TIFF) [file pone.0139034.s006.tiff]

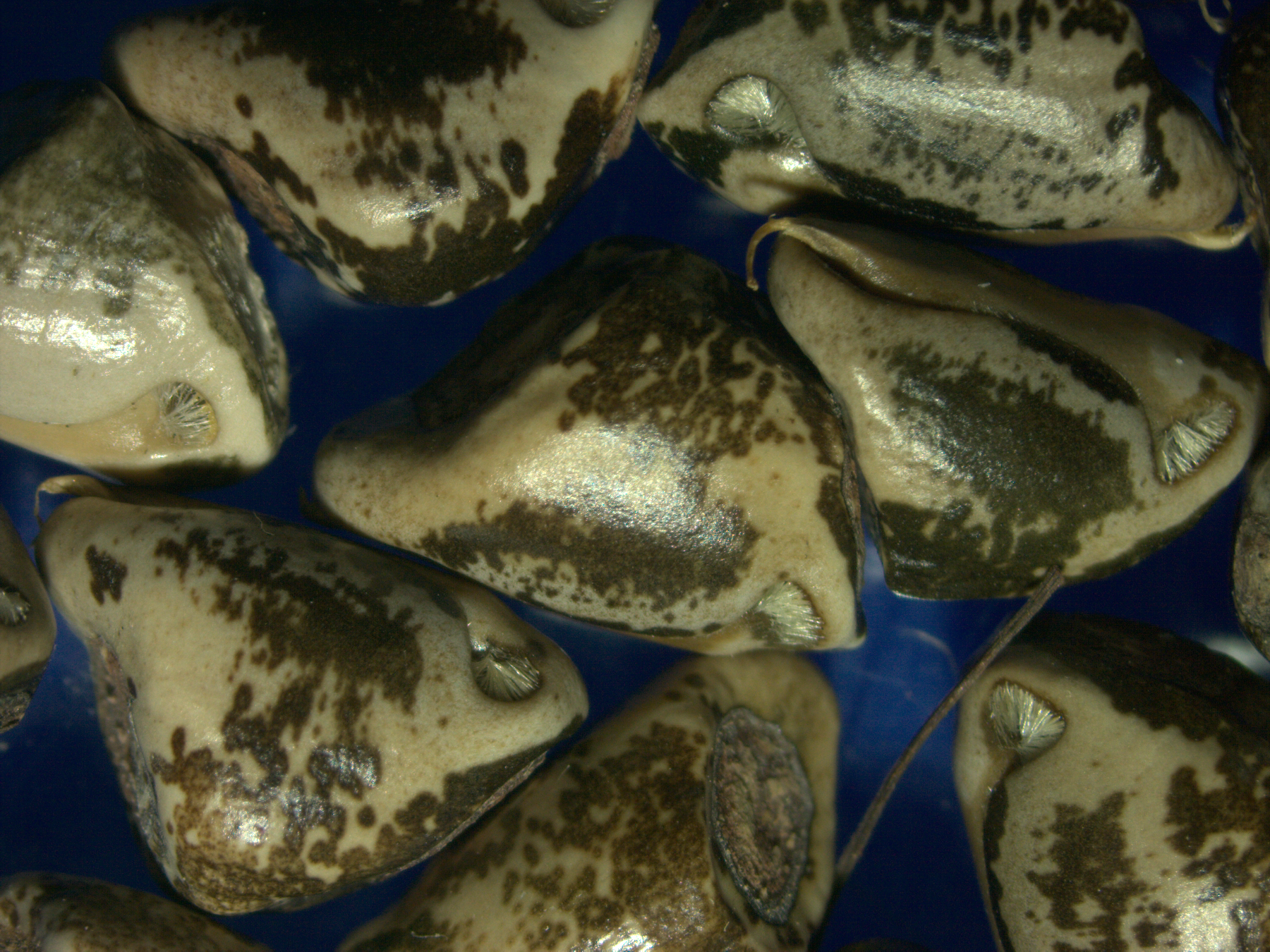

Supplement: S7 Fig — (TIFF) [file pone.0139034.s007.tiff]
